# Exploiting Nucleotide Composition to Engineer Promoters

**DOI:** 10.1371/journal.pone.0020136

**Published:** 2011-05-18

**Authors:** Manfred G. Grabherr, Jens Pontiller, Evan Mauceli, Wolfgang Ernst, Martina Baumann, Tara Biagi, Ross Swofford, Pamela Russell, Michael C. Zody, Federica Di Palma, Kerstin Lindblad-Toh, Reingard M. Grabherr

**Affiliations:** 1 Broad Institute, Cambridge, Massachusetts, United States of America; 2 Department of Biotechnology, University of Natural Resources and Life Sciences, Vienna, Austria; 3 Department of Medical Biochemistry and Microbiology, Uppsala University, Uppsala, Sweden; University of Minnesota, United States of America

## Abstract

The choice of promoter is a critical step in optimizing the efficiency and stability of recombinant protein production in mammalian cell lines. Artificial promoters that provide stable expression across cell lines and can be designed to the desired strength constitute an alternative to the use of viral promoters. Here, we show how the nucleotide characteristics of highly active human promoters can be modelled via the genome-wide frequency distribution of short motifs: by overlapping motifs that occur *infrequently* in the genome, we constructed contiguous sequence that is rich in GC and CpGs, both features of known promoters, but lacking homology to real promoters. We show that snippets from this sequence, at 100 base pairs or longer, drive gene expression *in vitro* in a number of mammalian cells, and are thus candidates for use in protein production. We further show that expression is driven by the general transcription factors TFIIB and TFIID, both being ubiquitously present across cell types, which results in less tissue- and species-specific regulation compared to the viral promoter SV40. We lastly found that the strength of a promoter can be tuned up and down by modulating the counts of GC and CpGs in localized regions. These results constitute a “proof-of-concept” for custom-designing promoters that are suitable for biotechnological and medical applications.

## Introduction

Artificially engineered promoter sequences have the potential for use in industrial and biotechnological applications, such as recombinant protein production of biopharmaceuticals. Some human proteins require mammalian cell lines for proper production, with e.g. the Chinese hamster ovary (CHO) cell line being a widely used system for EPO, Interferon-β, Factor VIII, IX, etc [1]–[3]. A crucial step in this process is the choice of an appropriate promoter, which, in the case of CHO, is complicated by the fact that the hamster genome is not available in its entirety. But even if it were, selecting existing mammalian promoters and screening them for activity is laborious and time consuming, and thus not viable on a large scale. A widely used alternative are viral promoters, such as the cytomegalovirus early promoter (CMV). However, these sequences have the disadvantage that they are fairly strong and fixed in strength, so that the stress imposed on the cells by producing foreign proteins sometimes leads to hampered growth and cell death. A more desirable solution are ‘artificial’ promoters, i.e. ‘made-up’ sequences that are not found in living organisms, that can be engineered to the required behaviour and expression levels. The more predictable these sequences, the easier it is to optimize a system for recombinant protein production. Avoiding viral sequences can also increase product safety. In this work, we devise methods to distil sequence features that allow for constructing such sequences, guided by observations gathered from real promoters, but without using their actual sequences.

The promoter is the genomic region around the transcription start site (TSS) of a gene, and acts as an essential component in gene regulation and transcription, its role being to interface with transcription factors (TFs) through protein-DNA binding. The TFs anchor the pre-initiation complex (PIC), specifying the exact point of initiation, and recruit RNA polymerase (Pol) II to start transcription [4]–[6]. A eukaryotic genome typically contains thousands of genes encoding TFs [7], which belong to several families. While the rest of the protein can vary considerably, the structure of their DNA-binding domains is often conserved [8]. As a consequence, many TFs, such as the homeodomain factors, exhibit sequence preference to similar sites, but the binding affinity varies on a continuous scale, sometimes involving a number of different, short (8 bp and less) DNA motifs [9]–[11]. To increase recognition specificity required for robust regulation beyond interaction between one single TF and the site it binds to, some TFs can form complexes that are pre-assembled prior to DNA interaction, requiring a specific organization of the promoter and exact spacing of elements that certain factors can bind to [12]–[13]. For example, it has been shown that the tandem orientation of two identical (or almost identical) binding sites in a promoter enables binding of a homo-dimer, to the effect that expression levels increase dramatically [14].

General transcription factors (GTFs), organized in complexes TFIIA, TFIIB, TFIID, TFIIE, TFIIF, TFIIH and TFIIJ, form a special class of TFs, in that they are ubiquitously present and both necessary and sufficient to enable Pol II transcription at significant levels, making these proteins desirable candidates as drivers of expression of artificial promoters. Only TFIIB and TFIID have been shown to exhibit sequence preference: the TATA-Binding Protein (TBP, a TFIID protein) is most well characterized and binds to the TATA-Box, thereby establishing the TSS 25–30 base pairs downstream of its location. However, only about 10% of human promoters rely on a TATA-Box [15], while the rest are reported to use other elements instead, such as the more degenerate initiator element [16]. While TBP and the TBP associated factors (TAFs) are generally attracted to motifs composed of the nucleotides Adenine (A) and Thymine (T), the TFIIB proteins prefer sequences rich in Cytosine (C) and Guanine (G), with the di-nucleotide CpG at its core (consensus sequence ‘SSRCGCC’ [17]). About half of the human promoters are rich in these features and are commonly characterized as CpG-islands [18], [19]. Highly active promoters show even higher enrichment, with 88% of promoters of genes present in IMR90 cells exhibiting this feature [20]. Since CpG is subject to spontaneous deamination, making it more vulnerable to mutation than other di-nucleotides, it is the most infrequent di-nucleotide genome-wide [21].

Designing artificial sequences that attract TFIIB and TFIID requires determining the features that capture the interactions between these proteins and the DNA. Generally, a complicating factor is the lack of a one-to-one relationship between TFs and exact instances of binding motifs, so that neither motif consensus of short sequences nor Position Weight Matrices (PWM) are ideal representations of binding sites [10]. Instead, we use the concept of *nucleotide composition*, rather than *sequence motifs*, where we define the term “nucleotide composition” as the frequency patterns of mono-nucleotides, di-nucleotides, tri-nucleotides etc. This composition varies over the human genome on a large scale, recognizable as isochores [22], as well as on a smaller scale as CpG islands [18]. CpG-containing motifs have been reported to be both necessary and sufficient to bind Pol II abundantly in more than one tissue to transcribe both housekeeping genes and genes with tissue-specific expression in multiple cell types [23]. Here, we show that artificial sequences that model the CpG richness of active promoters can drive gene expression *in vitro* in mammalian cells, as well as how expression levels depend on highly localized features in these sequences.

## Results

Highly active promoters exhibit nucleotide patterns that are different from the majority of the genome [21], most notably the abundance of GC and the di-nucleotide CpG. Assuming that these are features of active elements rather than a byproduct of other evolutionary mechanisms, it should be possible to construct artificial sequences that mimic these characteristics and function as promoters for *in vitro* expression. Here, we first recapitulate how highly active promoters (mostly associated with both housekeeping and strong tissue-specific genes) differ from the genome-wide distribution in GC and CpG, as well as in other di-nucleotides. We then exploit these “un-genomic” features by devising a measure that tracks the genome-wide frequency of each short motif: the less common genome-wide, the more likely it is to reflect properties of a promoter. We subsequently overlap a set of uncommon motifs to build “promoter-like” contiguous sequence, which allows for editing existing promoters, as well as to construct entirely artificial ones that work in a number of mammalian cells. We last show that TFIIB and TFIID bind to these promoters, which is reflected in their stability of expression level across multiple cell lines.

### Nucleotide distribution in highly active promoters

To capture a set of highly active promoters, we sequenced the mRNA of the most highly expressed genes in human cerebellum tissue. We constructed two cDNA libraries, one normalized and one un-normalized library (both filtered for poly-A tails), and sequenced both libraries on one lane of Illumina each, yielding a total of 2 billion base pairs in 71 bp long reads. We then assembled the reads from both libraries into contiguous transcripts using the transcriptome assembly program *Trinity* (Grabherr et al., in revision). The resulting assembly consists of 38 Mb of sequence, residing in 27,000 disjoint transcripts. We eliminated non-full-length transcript assemblies of less prominently expressed genes by requiring sequences to contain open reading frames of 500 bp or more, aligned the remaining sequences to the human genome, and selected only transcripts with the 5′ end falling within 50 bp of an annotated TSS [24]. This resulted in 1,746 highly expressed transcripts of known TSS, from each of which we defined 300 bp of promoter sequence (200 bp upstream of TSS and 100 bp downstream). These transcripts correspond to both tissue-specific genes as well as housekeeping ones: the two most highly expressed genes are the tissue-specific synaptosomal-associated protein 25 isoform (SNAP25), followed by housekeeping genes beta actin (ACTB), glial fibrillary acidic protein (GFAP), and ribosomal protein L3 (RPL3). The least expressed genes in this set have ∼300-fold less coverage than the top ones, and include haloacid dehalogenase-like hydrolase domain (HDHD1A), coiled-coil domain containing 134 (CCDC134), actin related protein 2/3 complex subunit 1B (ARPC1B), and amyloid beta (A4) precursor protein-binding (APBA3). In the complete set, ∼10% of the genes are up-regulated in cerebellum compared to other tissues according to Gene Expression Atlas 2 [25] (threshold = +1), indicating that this set captures a variety of genes involved in different cellular processes.

**Figure 1:**
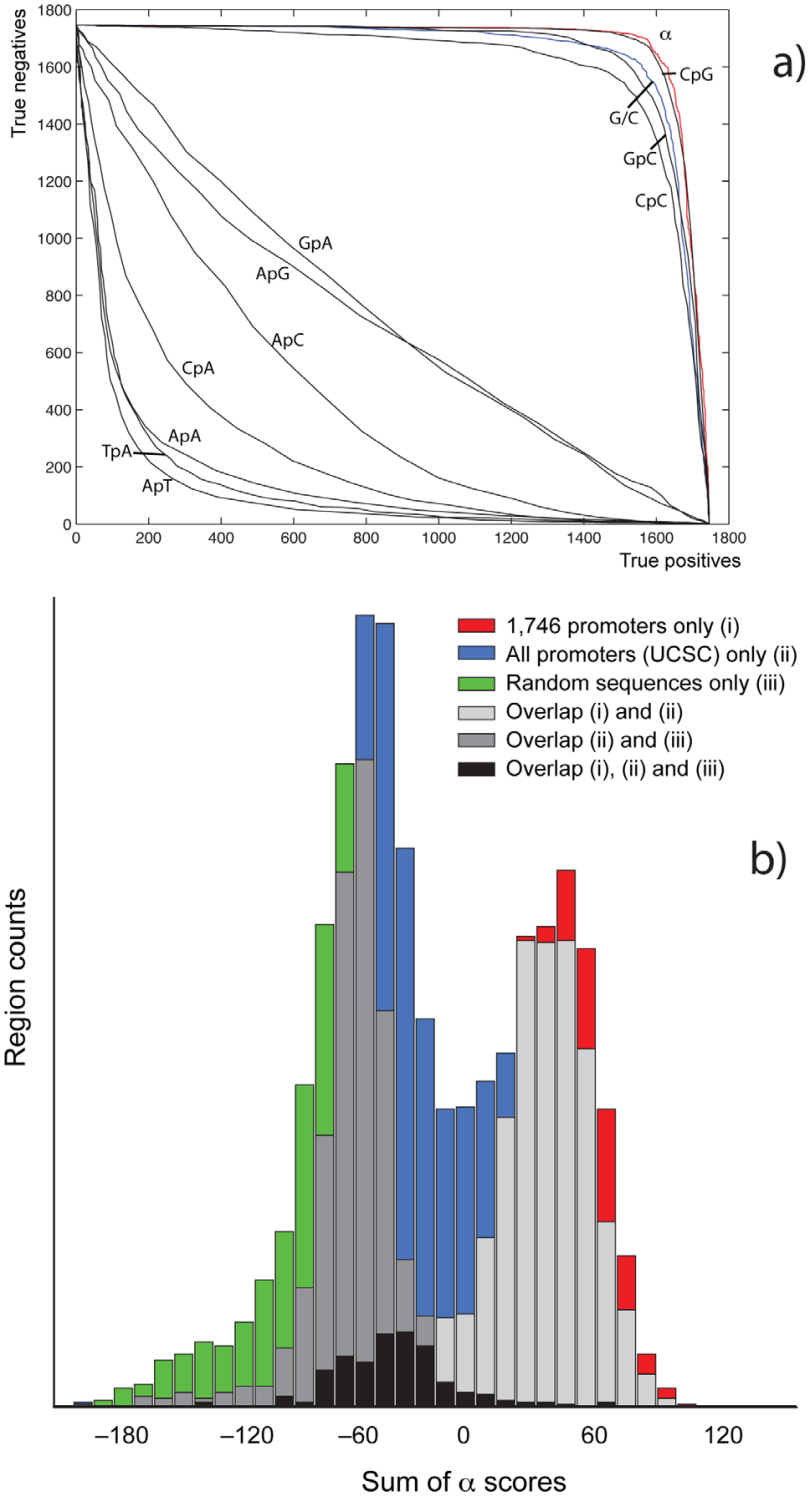
Receiver operator characteristics (ROC) of mono- and di-nucleotides (a), and histogram of α-score in promoter regions (b). (**a**) Several di-nucleotides are positive or negative classifiers distinguishing 1,746 experimentally confirmed promoters of genes active in human cerebellum tissue from random sequences, with CpG, GpC and CpC/GpG the strongest positive predictors and ApT, TpA, and ApA/TpT the strongest negative ones. Shown are also G/C content, as well as the α-scores summed over the promoter regions. (**b**) Shown is the distribution of regions over the sums of α-scores: 1,746 experimentally confirmed promoters of genes active in human cerebellum tissue in red (**i**), promoters chosen from the UCSC gene set [24] in blue (**ii**), randomly chosen sequences as negative control in green (**iii**), and the respective overlaps between the distributions in purple, dark chartreuse and black.

While only 127 (7.3%) of the 1,746 promoter sequences contain one or more instances of a TATA-Box in the correct orientation, the sequences are clearly distinct from the genome-wide average by their high G/C content (66% vs. 40% genome-wide) as well as the average frequency of CpGs (9% vs. 1% genome-wide). Figure 1a shows the receiver-operator characteristics for G/C content, as well as all di-nucleotides with their frequency as the discrimination threshold, distinguishing promoter sequences from a negative control set (1,746 randomly chosen, non-interspersed-repeat human sequences of equal length). The count of CpG's is the best discriminator: at a false positive (FP) prediction rate of 2%, the false negative (FN) rate is 13.5%, followed by GpC (FN = 25.1%), G/C content (FN = 30.6%) and CpC/GpC (FN = 53.9%). The di-nucleotides ApT, TpA and ApA/TpT act as negative predictors, with the remaining nucleotides being negative predictors as well, but at much lower sensitivity and specificity. CpG islands [18], [26], as binary classifiers, are specific discriminators, but much less sensitive than CG content and CpG, GpC and CpC/GpG, yielding a FN rate of 35% at an FP rate of 0.6%.

### Creating artificial sequences with nucleotide composition mimicking that found in highly active promoters

We defined a measure that tracks with G/C and CpG content (see Figure 1), incorporates contributions from other nucleotides, and can be computed for very short sequences so that these can be quantified as “promoter-like” on a sliding scale. To this end, we equate “promoter-like” with the extent to which they are “un-genomic”, i.e. unlike the majority of the genome. We computed a score (the “*α* score”, see Methods) over 12 consecutive base pairs (“12-mer”), based on the frequencies of di-nucleotides, tri-nucleotides etc. compared to the genome-wide expectation. To verify that this measure predominantly captures promoter-like features and not other ungenomic sequences, we scored all 12-mers in each of the 1,746 highly active promoters (see above) and used the sum of scores over the sequence as the discriminating function. We found that the receiver-operator characteristic of this method is close to, or slightly better than CpG counts (Figure 1a), with an FP rate of 2% on the control set (see above) yielding a FN rate of 11.5%. In addition to matching the discriminative power of CpG counts over hundreds of base pairs, *α* yields a potential spatial resolution of tens of nucleotides. Figure 1b shows a histogram of the 1,746 active promoter regions (i), and the distribution of regions based on 4,000 randomly selected *annotated TSS* [24] (ii), as well as the random control set for comparison (iii). Distribution (ii) is bi-modal with one peak coinciding with the experimentally found regions of active promoters (i), and a second peak more similar to the negative control (iii). We thus note that this method is not suitable to universally characterize all promoters, but rather models features of promoters associated with highly active genes.

To create longer, contiguous artificial promoter sequences, we began by calculating the *α* score for each possible 12-mer, including those not present in the human genome. We selected sequences from two quintiles: (a) the top 5% represent the most “un-genomic” (or promoter-like) 12-mers; and (b) the percentile between 45–50%, which contains 12-mers with di-, tri-nucleotide etc. frequencies close to the genomic median, representing more “normal” (or non-promoter-like) sequences. The 12-mers from each set were then independently assembled into “concatomers” (i.e. flattened 11-mer De Bruijn graphs [27] of maximum contiguous length to the extent that 11 bp overlaps exist within the 12-mer set). For the promoter-like concatomer, this resulted in a contiguous sequence of ∼160,000 base pairs, while the non-promoter-like concatomer was ∼180,000 base pairs long. Concatomer (a) is very rich in G/C (60%) and CpG (22%), and contains exact instances of the consensus of known binding sites, such as the TFIIB Recognition Element (BRE), TATA-Box, CAAT-Box, and Inr, while concatomer (b) is low in G/C (38%) and devoid of CpG's. Neither sequence has any homology to the human (or any other sequenced) genome over more than 18 base pairs.

### Modifying *in-vitro* promoter strength through sequence alteration

**Figure 2:**
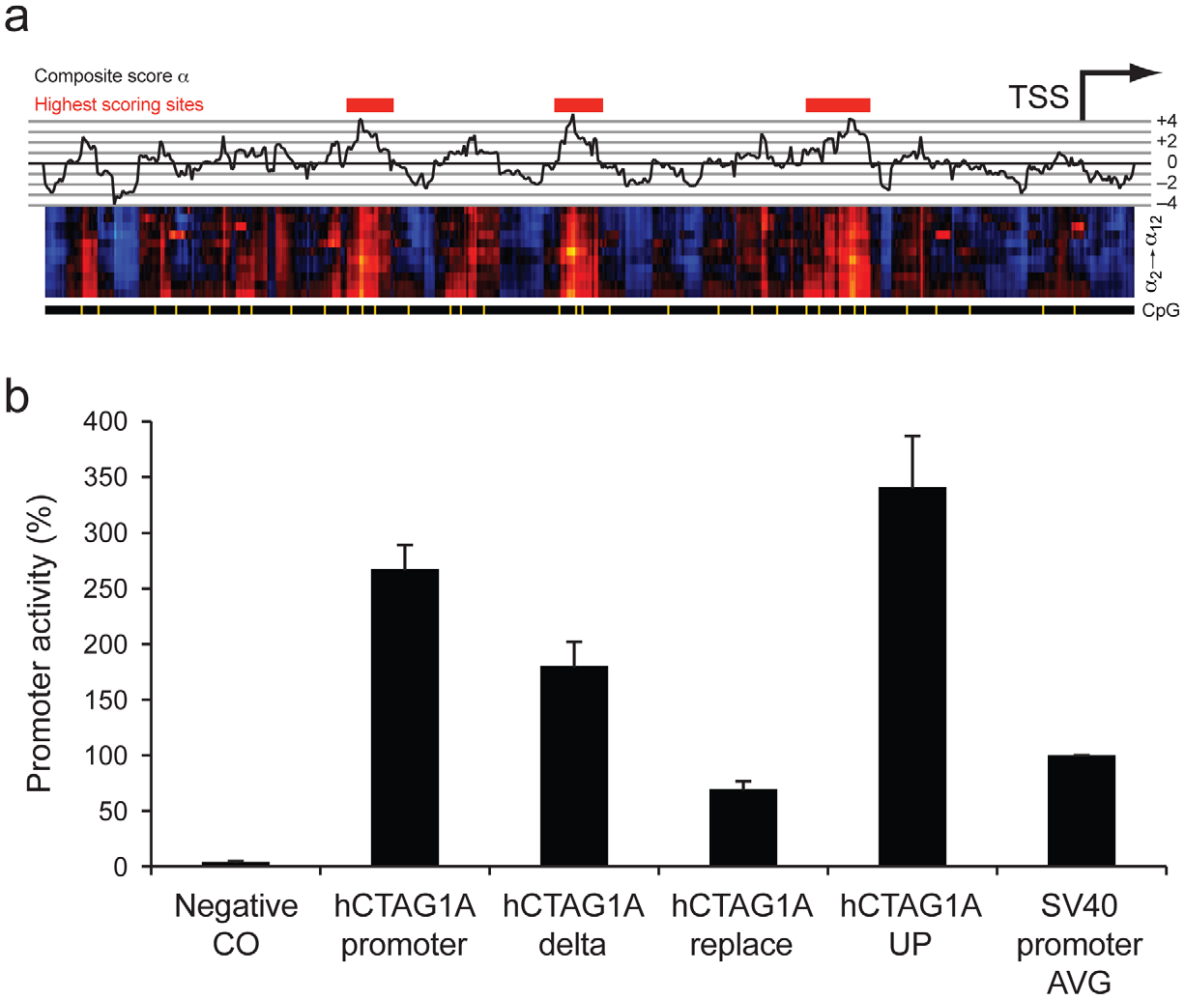
Human promoter CTAG1A and modified constructs. (**a**) The 535 base pair long promoter region of human gene CTAG1A is rich in CpGs and exhibits α-scores higher than the genomic distribution with pronounced peaks. Shown are the composite α_k_-scores (top), the individual α_k_-scores for different sizes of *k* in the middle graph (colour coded, blue = negative, red/orange = positive), and CpGs in yellow (bottom). The three strongest regions are marked by red bars. (**b**) In-vitro activity of the original CTAG1A promoter (hCTAG1A Promoter), the three strongest α-score regions deleted (hCTAG1A delta), the three strongest α-score regions replaced with sequences from the genomic concatomer (hCTAG1A replace), and the three strongest α-score regions replaced with sequences from the promoter-like concatomer (hCTAG1A UP). Also shown are results without any promoter (Negative CO) and the SV40 core promoter (SV40 Promoter AVG).

We tested whether we could modulate *in-vitro* activity by substituting selected sequences in a known promoter with sequences chosen from the promoter-like (for up-regulation) and non-promoter-like (for down-regulation) artificial constructs. As test case, we chose the promoter upstream of the TSS of the X-linked gene cancer/testis antigen 1A (CTAG1A), which exhibited strong *in-vitro* activity in human cell line HEK293. The CTAG1A promoter region contains three distinct regions of elevated *α* scores (red bars in Figure 2a), of lengths 23, 24 and 37 base pairs respectively (see Methods). As these regions have high *α* score, we expect that removal of these sequences will suppress promoter activity, as will replacement of these sequences with size-matched snippets from the non-promoter-like concatomer. Furthermore, we expect to be able to drive promoter activity by replacement of these (or any other regions within the promoter) with size-matched, but of higher *α* score, snippets from the promoter-like concatomer.

Figure 2b shows that this is in fact the case. Removal of the three regions without replacement (“hCTAG1A-delta”) suppresses *in-vitro* promoter activity in HEK293 cells relative to the original sequence, as does replacement with “non-promoter” sequences with *α* scores of approximately half the original (“hCTAG1A-replace”). Replacement with “promoter-like” sequences with an *α* score roughly twice that of the original (“hCTAG1A-UP”) increases activity beyond that of the original sequence. This indicates that highly localized changes in sequence composition can drive up- and down-regulation of *in-vitro* gene expression.

### Artificial promoter constructs drive *in-vitro* promoter activity

**Figure 3:**
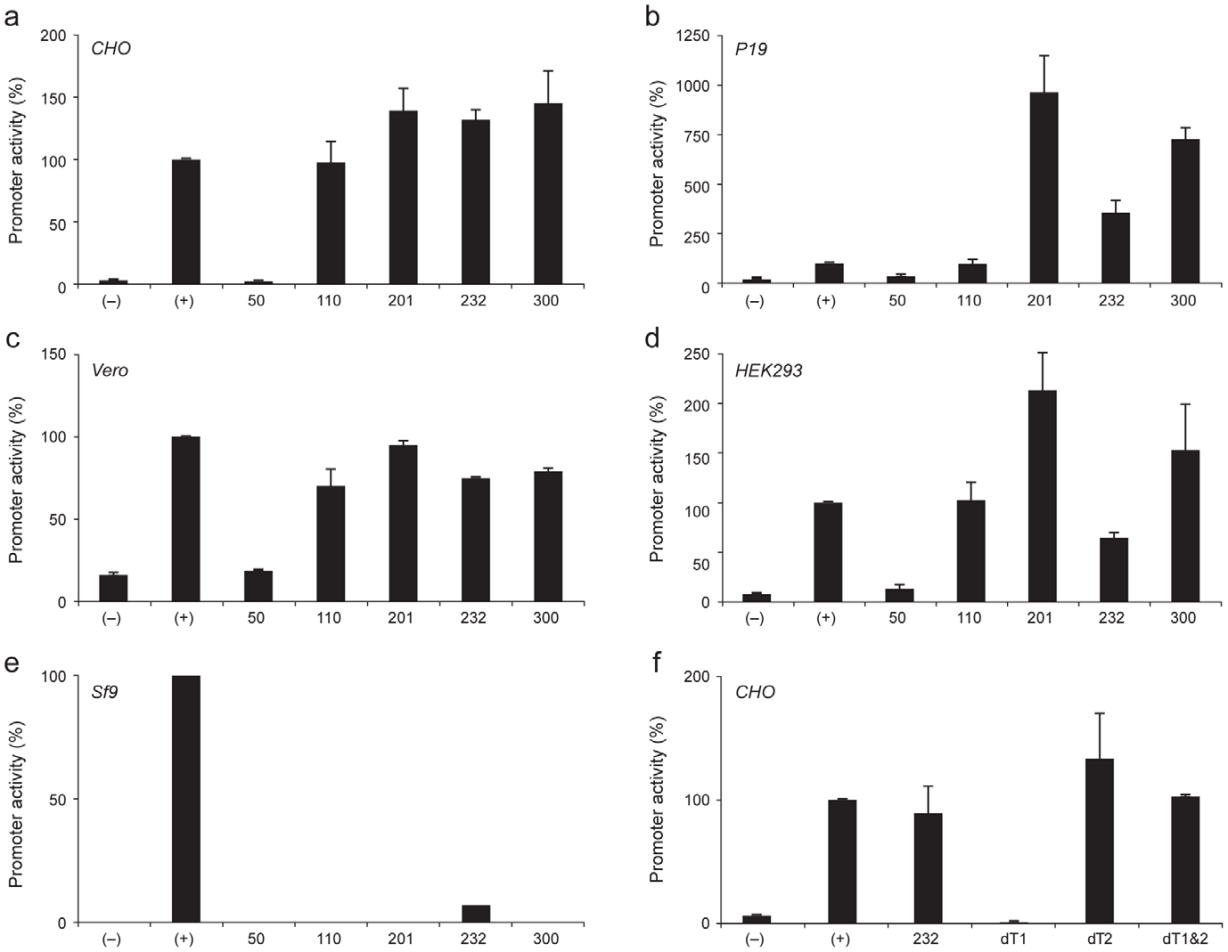
*In-vitro* promoter activity driven by artificial constructs. Artificial constructs ArS110, ArS300, ArS201 and ArS232 exhibit strong promoter activity driving a reporter gene (firefly luciferase, internally normalized by renilla luciferase) in mammalian cell lines: (**a**) CHO/hamster, (**b**) P19/mouse, (**c**) VERO/monkey, (**d**) HEK293/human, but not in (**e**) the insect cell line Sf9/army worm. Also shown are the negative control (−) and the SV40 core promoter activity (+). (**f**) TATA-boxes 1 (left) and 2 (right) were deleted from construct ArS232: deletion of TATA-box 1 only (dT1) results in lack of activity, deletion of TATA-box 2 (dT2) does not change expression levels, while deletion of both (dT1&2) results in slightly increased expression levels.

To create entirely artificial promoter constructs for *in-vitro* expression, we pulled sequences from the promoter-like concatomer using different criteria and of different lengths (50, 110, 200, 232 and 300 base pairs; see Methods) to create five artificial promoters and tested those for *in-vitro* promoter activity. With the exception of the shortest construct, all exhibited strong *in-vitro* promoter activity (as measured by firefly luciferase expression) in four mammalian cell lines: CHO (hamster ovary); P19 (mouse embryo); Vero (monkey kidney); and HEK293 (human kidney). Promoter strength of most constructs was comparable to, or exceeded activity of the SV40 core promoter, which is a routinely used viral promoter for recombinant protein expression in mammalian cell lines (Figure 3; see Methods). Notably, the longer constructs showed several fold higher activity in P19 than the SV40 core promoter, which shows only weak activity, indicating that the artificial sequences are rather unspecific to the cell type and/or which mammal the cells were derived from. By contrast, no activity was detected in the insect cell line Sf9 (*Spodoptera frugiperda*) for any of the constructs, suggesting fundamental differences in promoter mechanisms between insects and mammals (or perhaps, more widely, vertebrates).

All constructs contain at least one instance of a TATA-Box. In addition to two TATA-Boxes, ArS232 contains one perfect, and 12 imperfect (1 mismatch allowed) instances of BRE, and one Inr. To examine to what extent the TATA-box is needed to drive expression, we constructed three variations (Figure 3f): (i) removal TATA-Box2, which had no effect; (ii) removal of TATA-Box1, which completely reduced expression; and (ii) removal of both TATA-Box1 and TATA-Box2, which, again, showed strong expression (for sequences, see Methods). We explain this behaviour by TBP binding to the TATA-Box taking precedence over other GTFs in defining TSS ∼25 bp downstream of TATA-Box1, thus TBP binding to TATA-Box2 becomes irrelevant. In absence of TATA-Box1, TFIIB can define TSS through binding to CpG-rich motifs, possibly at Inr, but TBP bound to TATA-Box2 blocks transcription initiation, likely because of being located too close to the end, not allowing for a sufficiently long 5′ un-translated region (we also observe lack of activity in construct ArS50, which has a TATA-Box approximately as close to the end as ArS232). Subsequent removal of TATA-Box2 thus clears the way for transcription. We further note that all constructs including the original ArS232, with the exception of construct (ii), act as a promoter in both directions.

### General Transcription Factors bind to the artificial constructs

**Figure 4:**
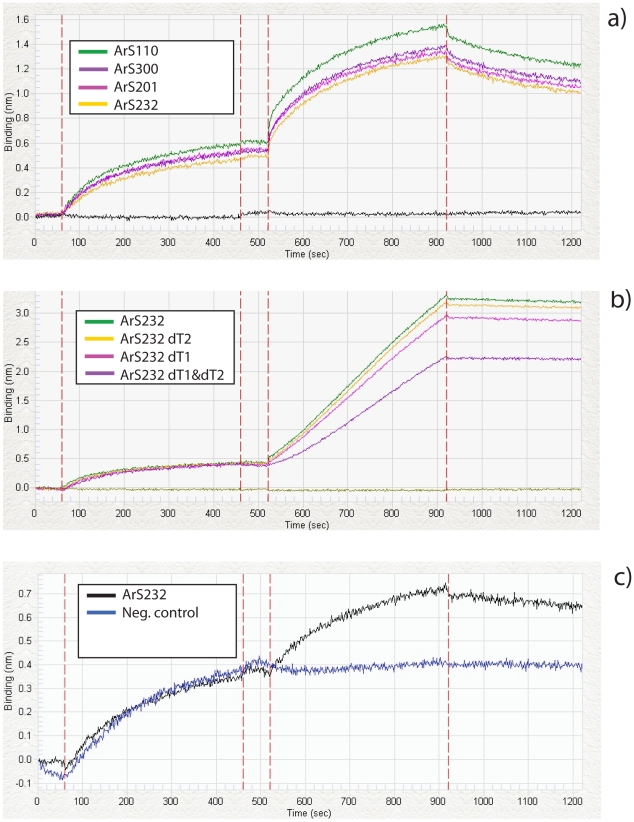
Binding affinity of artificial promoter constructs to the transcription factors TFIIB and TBP. The binding expressed as Δnm on the y-axis was monitored in real time as sec (x-axis), using the ForteBio Octet QK instrument. Binding was conducted in four phases: (i) loading of biotinylated DNA fragments to the streptavidin biosensor tip, (ii) washing in Kinetics Buffer, (iii) association of the transcription factor and (iii) dissociation of the transcription factor. (**a**) The promoter constructs ArS110, ArS201, ArS232 and ArS300 show similar binding affinities to the TFIIB protein. (**b**) The promoter constructs ArS232, ArS232 dT1, ArS232 dT2 and ArS232 dT12 exhibit sequence-specific binding to the TBP protein. ArS232 dT12 lacking two TATA-Boxes shows the lowest binding affinity compared to the other constructs. (**c**) TFIIB binding vs. a negative control, for which we chose a 85 bp long sequence from inside the coding region of the luciferase gene (pGL3-Basic Promoter Promega: 1314 bp–1399 bp).

The general transcription factors TFIIB and TBP bind to the artificial promoter constructs: we monitored real-time binding of TFIIB to constructs ArS110, ArS300, ArS201 and ArS232 through measurement of quantitative protein kinetics (Figure 4, see Methods). Figure 4a shows the respective real-time binding chart, clearly indicating TFIIB association with the constructs (seconds 520–920), followed by dissociation (for the binding constants, see Table 1). To quantify the role of TBP, we conducted a similar assay for constructs ArS232 and its derived constructs with the TATA-Box deletions (Figure 4b). Here, we found that the original sequence with both TATA-Boxes showed the highest level of association, while binding to construct ArS232 dT1&dT2, which lacks TATA-Boxes, was weaker in comparison. Unlike in case of TFIIB, binding increased linearly over time with little or no dissociation, possibly because of aggregation of TBP to protein already bound to DNA. We note that the linearity did not allow for computing appropriate K_D_ values, and that these readings are thus somewhat more difficult to interpret than the TFIIB binding results.

**Table 1:** Calculation of the binding constants *k*_on_, *k*_off_, and *K_D_* for the TFIIB binding assays.

DNA	K_D_ (M)	k_on_(1/Ms)	k_off_(1/s)
ArS 110	6.37E-08	3.03E+04	1.93E-03
ArS 201	7.76E-08	2.94E+04	2.28E-03
ArS 232	8.45E-08	2.73E+04	2.31E-03
ArS 300	6.46E-08	3.06E+04	1.98E-03

## Discussion

Artificially engineered promoter sequences have potential for use in industrial, biotechnological and medical applications involving recombinant protein production and gene therapy, since they can be designed to have different activities and be adapted to the specific requirements (strong or weak expression). The method proposed here yields constructs that appear less variable and species specifically regulated than the viral promoter SV40, a feature that would increase stability across cell types and conditions. By extension, it should be possible to build artificial test beds to determine the behavior of known binding sites, and subsequently design promoters that are targeted and regulated by specific transcription factors. Preliminary results already show promise that this is, in fact, the case, and future experiments will help expand our “vocabulary” of promoter elements, and to predict their effect on *in vitro* transcription depending of the abundance of the binding proteins that drive the promoter.

We do not address here how these results translate to *in vivo* expression, in presence of additional factors, such as methylation, degradation by miRNAs etc. While it might not be possible to accurately predict the behavior of artificially designed promoters in living organisms in the immediate short term, modifying short sub-sequences to adjust relative expression levels should be. In addition, emerging fields such as Synthetic Biology [28]–[30] that aim to create functioning, regulated systems from scratch in controlled environments might benefit from the results presented here.

This work might also be relevant for the study of expression regulation on a more general level: our findings suggest that different sequences can respond to transcription factors in very similar ways, even though they share no nucleotide sequence similarity; this would provide an explanation as to why promoters are generally not conserved across species over their entire length, but exhibit a pattern of conservation peaks and troughs [20]. Moreover, if promoter strength can easily be adjusted by changes in a few small regions, this mechanism provides an obvious approach for genome evolution, supplying natural selection with a fertile playground for experiments.

## Materials and Methods

### Computing the *α* score

The *α* score measures the “un-genomicness” of short (12 nucleotides long) sequences, taking into account the genome-wide frequencies of di-nucleotides, tri-nucleotides etc. within the sequence. Let *N* denote the number of k-mers (k consecutive nucleotides, k = 2, 3…12) in the genome, and *ϕ_k_^i^* the genome wide occurrence count of the k-mer starting at position *i* (using zero-based counting) in the 12 base pair (bp) long sequence, then the score *α_k_* is
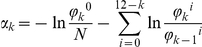
and the composite score for all *k*

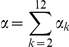



For existing promoter templates as well as the CTAG1A promoter, we computed the scores for all overlapping 12 bp long sequences within, and assigned the score to the base at position 6.

### Defining the regions for sequence substitution in the CTAG1A promoter

We defined the regions of the CTAG1A promoter for sequence substitution by selecting the three positions in the promoter with the highest composite *α* score, and extended the boundaries of each until its composite α score became negative, which yields a 2% false positive rate on the promoter template set (position 0 in Figure 1).

### Construction of synthetic promoter elements

Constructs ArS 50, 110, 201, 232 and 300 (50, 110, 201, 232, and 300 base pairs in length) were selected from the 160,000 nucleotide (nt) long promoter-like concatomer. For identification and spacing of TATA Boxes, we used a promoter prediction tool trained on fruit fly [31]. For ArS110, we selected three regions with high score hits, region one spans 24 consecutive nts, followed by 34 consecutive nts of region two, and region three is comprised of 52 nts. ArS 50 is a 50 bp fragment containing one of the program's top high-scores. ArS201 is derived from ArS300 after deletion events caused by plasmid amplification in *E. coli*. ArS200 and ArS232 consist of contiguous sequences out of the concatomer, containing 2 high-scores each. ArS300 consists of six 50 bp hits resulting in nine high-scores when stringed together. Constructs dT1, dT2 and dT12 comprise the sequence of ArS232, excluding the first, the second, and both TATA-Boxes respectively. The hCTAG1A promoter construct consists of the original 535 bp sequence upstream of the TSS of the X-linked human gene CTAG1A. Three more modified hCTAG1A constructs (CTAG1A-delta, CTAG1A-replace and CTAG1A-UP) were designed as described in the results section.

### Experimental quantification of promoter activity

All inserts have been assembled either by oligo synthesis (Sigma Aldrich, Austria) followed by annealing and PCR or by gene synthesis (Geneart, Germany) and cloning into the reporter vector pGL3 Basic (Promega, Madison, Wisconsin) upstream of a firefly luciferase gene. After propagation in *E. coli* and purification using NucleoSpin Extrakt II (Macherey-Nagel, Germany) all plasmids were sequenced to confirm the original sequence. In case of CHO dhfr-, HEK293 and P19, 4×10∧6 cells were transfected with 10 µg of the firefly luciferase plasmids and co-transfected with 1 µg of the Renilla luciferase reporter vector pRL-SV40 (Promega, Madison, Wisconsin) as an internal standard using Amaxa's Nucleofector Kit V (Lonza, Switzerland) according to the manufacturer's instructions. 3×10∧5 VERO and MDCK cells were transfected with 1 µg DNA of the firefly luciferase constructs and co-transfected with 0,25 µg pRL-SV40 using Dreamfect Gold and CombiMag (OZ Biosciences, Marseille, France). 3×10∧6 *Sf*9 cells were transfected with 1 µg of the firefly luciferase constructs using Cellfectin II (Invitrogen, Carlsbad, CA) according to the manufacturer's instructions. Instead of the SV40 promoter the baculovirus derived immediate early promoter OplE2, which is active in insect cells was used for the positive control. Luciferase expression was measured 48 h post transfection on a Synergy 2 microplate reader (Biotek, Vermont, USA) with the Gen5 software using the Dual-Glo luciferase assay system (Promega, Madison, Wisconsin). To normalize transfection efficiency, promoter activities are expressed as the ratio of firefly and Renilla luciferase activity. The pGL3-Promoter plasmid (Promega, Madison, Wisconsin), containing the SV40 promoter served as positive control. The promoter activity of this viral promoter was set to 100%. All other measurements refer to this value within the same cell-line. The promoterless pGL3-Basic Vector (Promega, Madison, Wisconsin) was used as a negative control.

### Binding assays

For the identification of sequence-specific DNA binding of the transcription factors TFIIB and TBP the binding kinetics were measured by biolayer interferometry on an Octet QK instrument (ForteBio Inc.), which provides continuous real-time display of biomolecular interactions. Streptavidin biosensors were loaded with biotinylated DNA fragments (25 µg/ml) of the promoter constructs ArS110, ArS201, ArS232 and ArS300, or with the promoter constructs ArS232, ArS232 dT1, ArS232 dT2 and ArS232 dT12, generated by PCR amplification using 5′ biotinylated primer (Sigma-Aldrich). Binding was conducted in 1× Kinetics Buffer (ForteBio Inc.) with a protein concentration of 285 nM for TBP (catalog # ab81897, Abcam) and 270 nM for TFIIB (catalog # ab1898, Abcam). Kinetic parameters (k_on_ and k_off_) and affinities (K_D_) were calculated using the Octet Data Analysis Software Version 6.3.

### Construct sequences

**>ArS 50**

ACGCACGCGGTATAAACGCGCGACCTATTCGCGACCGTATAGCGACCGGA

**>ArS 110**

CTACGCCGCGTAAATATCGCGCGCTAACGGTGCGCGTTAAAACGCCGACGCGTCATAAAGCGCCGGCGTATAAGCGCGCCGTACGTCGTCGAACCACGTTAGTCCGGACC

**>ArS 201**

AACGGTGCGCGTTAAAACGGCCGACGCGTCATAACCGCGACTCGTCGACGCAGCGCCGGCGTATAAGCGCGCCGTACGTCAACCGTCGACGTTAGTCCGACGATCGCGGCGTCTATACGCCGCGTCAATCGCGCGCGGTTCAACGTCGCGCTACGGGCGCGTATAAGTCGCGCGTATGGACCGCGTACGTCCTACGAGCGT

**>ArS 232**

TCGACGCGCGTATAACACGCGAGCGGTTCGAACGTTGGCGCGCTAACGCGAGTCGTACGCCCGTCAACGCGGATCAATCGCGCGACTTGTGCGCGACGTTAGACCGCCGATCGTCAAGCGCCGATCGGTAATCGGACGATTCGGATACGCGAGTTCGGACGTACGAGCGTGATACGGCGCGTAACGGTGCGCGTTAAAACGCCGACGCGTCATAACCGCGACTCGTCGACGC

**>ArS 300**

AACGGTGCGCGTTAAAACGCCGACGCGTCATAACCGCGACTCGTCGACGCAGCGCCGGCGTATAAGCGCGCCGTACGTCAACCGTCGACGTTAGTCCGACGATCGCGGCGTCTATACGCCGCGTCAATCGCGCGCGGTTCAACGTCGCGCTACGGGCGCGTATAAGTCGCGCGGTTAATACGCGCGGTGTACGCGGATGCCGGGGTCGCGTATAATCGGCGCGTATACCTCGCGCGTATACGCGGCGTATTACGGCCGCGTATAATTCGCGCGTATGGACCGCGTACGTCCTACGAGCGT

**>CTAG1A_original**

TCTCAGAGAGAAGGTCAGGGCCCACGAGGATGCGGAGGCAGAGAGGCTGCAGGAAGTTCCGCCCCCTGGCGTGAGATGGGCAGCCCGGGATCCTCAGGGCGCCTGCGCACAGGGGCCCTACTTCCGGCCCTGGGAGACCCCGAGTGAGCCC**CGGAGCACGTGACCGGTTCTCAC**CAACCCCGCCCCTCCCCAAGAGAGCCCGGGCCGGAAGGTGGCCGCAATGCCAGCTTGGACCCCTCACCCCTGAGC**AGCCGGCTGTCCGCCGGACCCCTG**TCCCGGGAGCCCTGCAGGGAGTCAGGCACTGCGGGGCCCAGCCTGTCCCATCCCCCGGGTCTCCCTCACATCGAGGAGCAAGACGGGCCTGGGAACACGGGGCCGG**GACTGTGCGGCCATCGTCCCGGACCCTGCCTGCCCTG**TCCGTCCTTGGGGGAGCGCCCAGGACAGACfCCCGGGGGGCAGGCCTCTAfACTGGGCTCAGCAGCCTCCGTCCCTGTCCTGGTCGCCCAGCTGGTGGGGTAGCTGGAACTGCATGTCTGG

**>CTAG1A_replace**

TCTCAGAGAGAAGGTCAGGGCCCACGAGGATGCGGAGGCAGAGAGGCTGCAGGAAGTTCCGCCCCCTGGCGTGAGATGGGCAGCCCGGGATCCTCAGGGCGCCTGCGCACAGGGGCCCTACTTCCGGCCCTGGGAGACCCCGAGTGAGCCC**TTACCTAAAACAGCCCAAAAGAG**CAACCCCGCCCCTCCCCAAGAGAGCCCGGGCCGGAAGGTGGCCGCAATGCCAGCTTGGACCCCTCACCCCTGAGC**CCACCACCACCTCCACCACCACTG**TCCCGGGAGCCCTGCAGGGAGTCAGGCACTGCGGGGCCCAGCCTGTCCCATCCCCCGGGTCTCCCTCACATCGAGGAGCAAGACGGGCCTGGGAACACGGGGCCGG**CCAAAGAAGCCCAAAAAGGCCCAGGAAACCCAAACTT**TCCGTCCTTGGGGGAGCGCCCAGGACAGACCCCGGGGGGCAGGCCTCTAACTGGGCTCAGCAGCCTCCGTCCCTGTCCTGGTCGCCCAGCTGGTGGGGTAGCTGGAACTGCATGTCTGG

**>CTAG1A_delta**

TCTCAGAGAGAAGGTCAGGGCCCACGAGGATGCGGAGGCAGAGAGGCTGCAGGAAGTTCCGCCCCCTGGCGTGAGATGGGCAGCCCGGGATCCTCAGGGCGCCTGCGCACAGGGGCCCTACTTCCGGCCCTGGGAGACCCCGAGTGAGCCCCAACCCCGCCCCTCCCCAAGAGAGCCCGGGCCGGAAGGTGGCCGCAATGCCAGCTTGGACCCCTCACCCCTGAGCTCCCGGGAGCCCTGCAGGGAGTCAGGCACTGCGGGGCCCAGCCTGTCCCATCCCCCGGGTCTCCCTCACATCGAGGAGCAAGACGGGCCTGGGAACACGGGGCCGGTCCGTCCTTGGGGGAGCGCCCAGGACAGACCCCGGGGGGCAGGCCTCTAACTGGGCTCAGCAGCCTCCGTCCCTGTCCTGGTCGCCCAGCTGGTGGGGTAGCTGGAACTGCATGTCTGG

**>CTAG1A_up**

TCTCAGAGAGAAGGTCAGGGCCCACGAGGATGCGGAGGCAGAGAGGCTGCAGGAAGTTCCGCCCCCTGGCGTGAGATGGGCAGCCCGGGATCCTCAGGGCGCCTGCGCACAGGGGCCCTACTTCCGGCCCTGGGAGACCCCGAGTGAGCCC**CGTTTGACGGACGCCGTTCGCAGT**CAACCCCGCCCCTCCCCAAGAGAGCCCGGGCCGGAAGGTGGCCGCAATGCCAGCTTGGACCCCTCACCCCTGAGC**CGGAGCACGTGACCGGTTCTCAC**TCCCGGGAGCCCTGCAGGGAGTCAGGCACTGCGGGGCCCAGCCTGTCCCATCCCCCGGGTCTCCCTCACATCGAGGAGCAAGACGGGCCTGGGAACACGGGGCCGG**ATCGCGCAGCGATCGACGCCGGATCAACGCGATACGG**TCCGTCCTTGGGGGAGCGCCCAGGACAGACCCCGGGGGGCAGGCCTCTAACTGGGCTCAGCAGCCTCCGTCCCTGTCCTGGTCGCCCAGCTGGTGGGGTAGCTGGAACTGCATGTCTGG

### Alignment of deletion mutants dT1, dT2 dT12 and parental sequence ArS232

232 TCGACGCGCG**TATAA**CACGCGAGCGGTTCGAACGTTGGCGCGCTAACGCGAGTCGTACGC 60

dT1 TCGACGCGCG-----CACGCGAGCGGTTCGAACGTTGGCGCGCTAACGCGAGTCGTACGC 55

dT2 TCGACGCGCG**TATAA**CACGCGAGCGGTTCGAACGTTGGCGCGCTAACGCGAGTCGTACGC 60

dT12 TCGACGCGCG-----CACGCGAGCGGTTCGAACGTTGGCGCGCTAACGCGAGTCGTACGC 55

********** *********************************************

232 CCGTCAACGCGGATCAATCGCGCGACTTGTGCGCGACGTTAGACCGCCGATCGTCAAGCG 120

dT1 CCGTCAACGCGGATCAATCGCGCGACTTGTGCGCGACGTTAGACCGCCGATCGTCAAGCG 115

dT2 CCGTCAACGCGGATCAATCGCGCGACTTGTGCGCGACGTTAGACCGCCGATCGTCAAGCG 120

dT12 CCGTCAACGCGGATCAATCGCGCGACTTGTGCGCGACGTTAGACCGCCGATCGTCAAGCG 115

************************************************************

232 CCGATCGGTAATCGGACGATTCGGATACGCGAGTTCGGACGTACGAGCGTGATACGGCGC 180

dT1 CCGATCGGTAATCGGACGATTCGGATACGCGAGTTCGGACGTACGAGCGTGATACGGCGC 175

dT2 CCGATCGGTAATCGGACGATTCGGATACGCGAGTTCGGACGTACGAGCGTGATACGGCGC 180

dT12 CCGATCGGTAATCGGACGATTCGGATACGCGAGTTCGGACGTACGAGCGTGATACGGCGC 175

************************************************************

232 GTAACGGTGCGCG**TTAAAA**CGCCGACGCGTCATAACCGCGACTCGTCGACGC 232

dT1 GTAACGGTGCGCG**TTAAAA**CGCCGACGCGTCATAACCGCGACTCGTCGACGC 227

dT2 GTAACGGTGCGCG------CGCCGACGCGTCATAACCGCGACTCGTCGACGC 226

dT12 GTAACGGTGCGCG------CGCCGACGCGTCATAACCGCGACTCGTCGACGC 221

************* *********************************
